# Subarachnoid Pneumocephalus Following Endoscopic Transsphenoidal Surgery

**DOI:** 10.5811/cpcem.64485

**Published:** 2026-09-08

**Authors:** Willie Dong, Roger Rothenberg

**Affiliations:** Temple University Hospital, Department of Emergency Medicine, Philadelphia, Pennsylvania

**Keywords:** pneumocephalus, subarachnoid, case image

## Abstract

**Case Presentation:**

A 26-year-old man with past medical history of prolactinoma presented two weeks status post endoscopic transsphenoid prolactinoma excision with a positional headache and cerebrospinal fluid rhinorrhea; he was found to have diffuse subarachnoid air.

**Discussion:**

There are few reports of subarachnoid pneumocephalus in the emergency medicine literature. Pneumocephalus, particularly tension pneumocephalus, typically occurs in the setting of traumatic skull fractures. However, emergency physicians should be aware of subarachnoid pneumocephalus in the setting of endoscopic skull base surgeries.

## CASE PRESENTATION

A 26-year-old man with past medical history of prolactinoma status post endoscopic transsphenoid excision with neurosurgery, about two weeks prior, presented to the emergency department (ED) for headache. The patient awoke on the morning of presentation with a sharp bilateral headache that worsened when sitting up. He also reported clear nasal drainage with a salty taste and postnasal drainage into the posterior oropharynx. He denied fever or other infectious symptoms. He had received postsurgical discharge instructions to present to the ED for headache or neurological symptoms. His vital signs on arrival were within normal limits. He was in no acute distress. Neurologic examination was nonfocal aside from a baseline temporal hemianopsia of the left eye attributable to his prolactinoma. Noncontrast computed tomography (CT) of the head demonstrated diffuse subarachnoid air (Images 1–3).

In the ED, neurosurgery was consulted following CT acquisition and prior to formal radiology interpretation because of the abnormal imaging findings. The patient was subsequently admitted to the neuro-intensive care unit, and a lumbar drain was placed for persistent cerebrospinal fluid (CSF) leak. On hospital day 4 he returned to the operating room for an endoscopic CSF leak repair. The lumbar drain was removed on hospital day 10. Recurrence of CSF rhinorrhea on hospital day 14 necessitated placement of a second lumbar drain on hospital day 17 and repeat endoscopic CSF leak repair on day 19. The second drain was removed on hospital day 25. He was discharged on hospital day 27 and continues outpatient follow-up without recurrent complications.

## DISCUSSION

Pneumocephalus is an uncommon finding in the ED, with most cases occurring in the setting of skull fractures.^1^ There are few reports of pneumocephalus in the emergency medicine literature, and only one prior report of subarachnoid pneumocephalus following an epidural steroid injection.^2^–^10^ Reports of pneumocephalus following endoscopic skull base surgery are similarly rare and largely confined to the otolaryngology and neurosurgical literature.^11^–^14^ To our knowledge, no prior cases of diffuse subarachnoid pneumocephalus following endoscopic skull base surgery have been reported in the emergency medicine literature.

*CPC-EM Capsule*

What do we already know about this clinical entity?

*Pneumocephalus is uncommon and usually follows trauma or skull fracture. Tension pneumocephalus is a neurosurgical emergency.*

What makes this presentation of disease reportable?

*Diffuse subarachnoid pneumocephalus after endoscopic transsphenoidal surgery has not been reported in emergency medicine literature.*

What is the major learning point?

*Positional headache and salty rhinorrhea after skull-base surgery should raise suspicion for pneumocephalus and cerebrospinal fluid leak.*

How might this improve emergency medicine practice?

*Early recognition of red-flag symptoms and computed tomography findings can expedite neurosurgical consultation and definitive management.*

Clinical diagnosis of pneumocephalus can be challenging because patients may be asymptomatic or present with nonspecific symptoms, such as headache, that overlap with many other diagnoses.^1^ In contrast, tension pneumocephalus is a neurosurgical emergency that may present with signs of elevated intracranial pressure, deteriorating mental status, focal neurologic deficits, cerebrospinal fluid rhinorrhea, or seizures. Computed tomography remains the gold standard for diagnosis of pneumocephalus and tension pneumocephalus. Classic CT findings suggestive of tension physiology include the “Mount Fuji sign” and “peaking sign” in the setting of subdural pneumocephalus or the “air bubble sign” for subarachnoid pneumocephalus.^7^,^8^,^10^–^14^

As demonstrated in this case, the air bubble sign is characterized by multiple air bubbles distributed throughout multiple cisterns and the subarachnoid space. Consistent with previously described “red flag” headache features, this patient reported a positional headache^15^ and recent surgical intervention, prompting early neurosurgical consultation and advanced imaging. As with subarachnoid hemorrhage, emergency physicians should be able to identify this diagnosis on CT, and care should not be delayed for radiologist interpretation. Emergency physicians should maintain a high index of suspicion for pneumocephalus in postoperative patients with skull base surgeries. This consideration is particularly important in postoperative skull base surgery patients presenting with concerning features such as a positional headache, salty or metallic-tasting rhinorrhea, maximal headache on awakening, and worsening pain with Valsalva. Additional diagnoses that may present similarly, including subarachnoid hemorrhage, spontaneous intracranial hypotension, infection, and malignancy, should also be considered.

## Figures and Tables

**Image 1:**
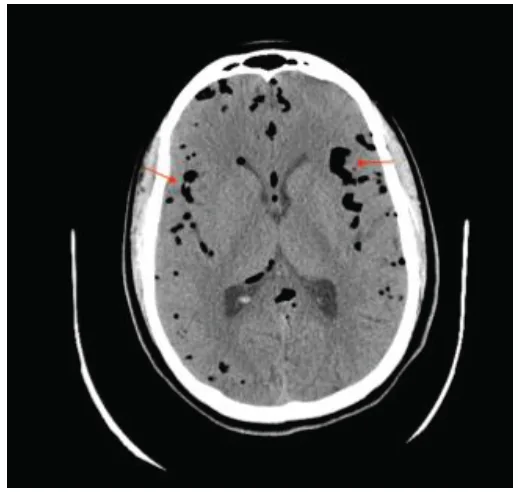
Axial view from head computed tomography without contrast demonstrating subarachnoid air. Red arrows indicate collections of hypoattenuation, representing air in the subarachnoid space.

**Image 2:**
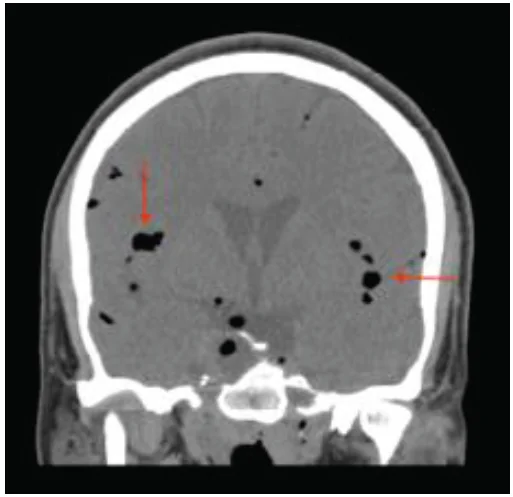
Coronal view from head computed tomography without contrast demonstrating subarachnoid air. Red arrows indicate collections of hypoattenuation, representing air in the subarachnoid space.

**Image 3:**
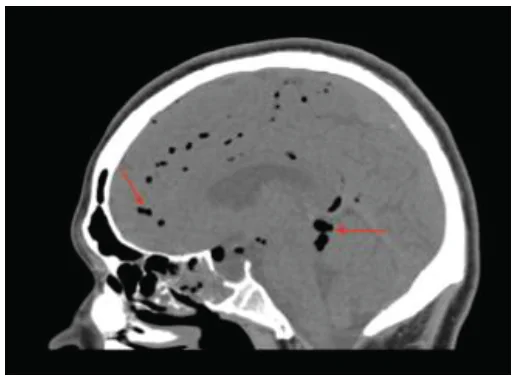
Sagittal view from head computed tomography without contrast demonstrating subarachnoid air. Red arrows indicate collections of hypoattenuation, representing air in the subarachnoid space.

## References

[1] Das JM, Munakomi S, Bajaj J (2025). Pneumocephalus.

[2] Chacko J, Levis K, Hahn B (2018). Pneumocephalus after epidural injection. J Emerg Med.

[3] Beaumont R, Anderson R, Carlson JN (2021). An unusual case of spontaneous nontraumatic pneumocephalus in the setting of remote craniofacial surgery. Am J Emerg Med.

[4] Simoulidou E, Georgopoulou V, Kalmoukos P (2023). Pneumocephalus; a rare cause of coma. Am J Emerg Med.

[5] Wu YH, Yeh IJ, Liu KT (2018). Spontaneous pneumocephalus and subdural hemorrhage after sneezing. Am J Emerg Med.

[6] Chen J, Wang SA, Huang CY (2023). Spontaneous pneumocephalus: a case report with a literature review. J Emerg Med.

[7] L’Hommedieu LM, Dingeldein MW, Tomei KL (2018). Acute management of tension pneumocephalus in a pediatric patient: a case report. J Emerg Med.

[8] Parish JM, Driscoll J, Wait SD (2020). Delayed traumatic tension pneumocephalus: a case report. J Emerg Med.

[9] Shah AK, Bilko A, Takayesu JK (2016). Epidural steroid injection complicated by intrathecal entry, pneumocephalus, and chemical meningitis. J Emerg Med.

[10] Simmons J, Luks AM (2013). Tension pneumocephalus: an uncommon cause of altered mental status. J Emerg Med.

[11] Jundt JS, Bohnen A, Galbraith BS (2022). Tension pneumocephalus: case report and review. Int J Oral Maxillofac Surg.

[12] Biju RD, Wu J, Hussain Z (2020). Tension pneumocephalus after skull base surgery. a case report and review of literature. J Clin Neurosci.

[13] Martínez-Capoccioni G, Serramito-García R, Cabanas-Rodríguez E (2014). Tension pneumocephalus as a result of endonasal surgery: an uncommon intracranial complication. Eur Arch Otorhinolaryngol.

[14] Li W, Liu Q, Lu H (2020). Tension pneumocephalus from endoscopic endonasal surgery: a case series and literature review. Ther Clin Risk Manag.

[15] Do TP, Remmers A, Schytz HW (2019). Red and orange flags for secondary headaches in clinical practice: SNNOOP10 list. Neurology.

